# Detailed Distribution Map of Absorbed Dose Rate in Air in Tokatsu Area of Chiba Prefecture, Japan, Constructed by Car-Borne Survey 4 Years after the Fukushima Daiichi Nuclear Power Plant Accident

**DOI:** 10.1371/journal.pone.0171100

**Published:** 2017-01-27

**Authors:** Kazumasa Inoue, Moeko Arai, Makoto Fujisawa, Kyouko Saito, Masahiro Fukushi

**Affiliations:** 1 Department of Radiological Sciences, Graduate School of Human Health Sciences, Tokyo Metropolitan University, Arakawa-ku, Tokyo, Japan; 2 Department of Radiological Technology, Faculty of Health Sciences, Nihon Institute of Medical Science, Iruma-gun, Saitama, Japan; University of Liverpool, UNITED KINGDOM

## Abstract

A car-borne survey was carried out in the northwestern, or Tokatsu, area of Chiba Prefecture, Japan, to make a detailed distribution map of absorbed dose rate in air four years after the Fukushima Daiichi Nuclear Power Plant accident. This area was chosen because it was the most heavily radionuclide contaminated part of Chiba Prefecture and it neighbors metropolitan Tokyo. Measurements were performed using a 3-in × 3-in NaI(Tl) scintillation spectrometer in June 2015. The survey route covered the whole Tokatsu area which includes six cities. A heterogeneous distribution of absorbed dose rate in air was observed on the dose distribution map. Especially, higher absorbed dose rates in air exceeding 80 nGy h^-1^ were observed along national roads constructed using high porosity asphalt, whereas lower absorbed dose rates in air were observed along local roads constructed using low porosity asphalt. The difference between these asphalt types resulted in a heterogeneous dose distribution in the Tokatsu area. The mean of the contribution ratio of artificial radionuclides to absorbed dose rate in air measured 4 years after the accident was 29% (9–50%) in the Tokatsu area. The maximum absorbed dose rate in air, 201 nGy h^-1^ was observed at Kashiwa City. Radiocesium was deposited in the upper 1 cm surface layer of the high porosity asphalt which was collected in Kashiwa City and the environmental half-life of the absorbed dose rate in air was estimated to be 1.7 years.

## Introduction

The environmental radiation levels in eastern Japan were dramatically changed after the Fukushima Daiichi Nuclear Power Plant (F1-NPP) accident in March 2011. According to the UNSCEAR 2013 report [1], the released total amounts of artificial radionuclides were estimated to be 6–20 PBq of ^137^Cs and 100–500 PBq of ^131^I and they are about 20% and 10% of the respectively estimated amounts emitted in the 1986 Chernobyl accident. Since the F1-NPP accident, distributions of absorbed dose rates, affected by artificial radionuclides, have been observed by public officials and researchers [2–7]. In the most extensive surveys, the Japanese government has carried out air- and car-borne surveys centered on Fukushima Prefecture at regular intervals, and the distribution maps of dose equivalent rate have been made available on the website of the Nuclear Regulation Authority, Japan [8].

**Fig 1:**
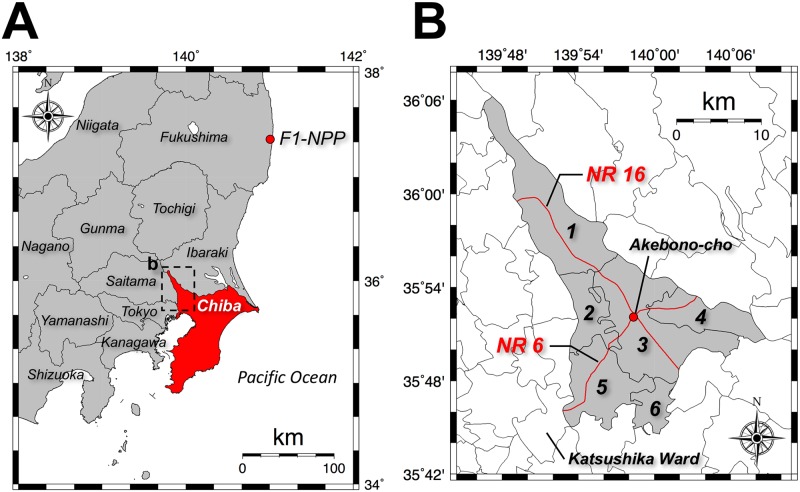
The location of Chiba Prefecture relative to the Fukushima Daiichi Nuclear Power Plant (A) and the detailed location of the Tokatsu area in the northwestern part of Chiba Prefecture (B) which includes six cities: 1, Noda City; 2, Nagareyama City; 3, Kashiwa City; 4, Abiko City; 5, Matsudo City; and 6, Kamagaya City.

The Tokatsu area is located in the northwestern part of Chiba Prefecture (Fig 1) and it includes the six cities of Noda, Nagareyama, Kashiwa, Abiko, Matsudo and Kamagaya. Within Chiba Prefecture, this area received the most radionuclide contamination from the F1-NPP accident [8]. Initially, the Japanese government did not provide support for environmental surveys in these cities because they were located outside Fukushima Prefecture; support was begun 6 months after the accident. Therefore, in response to requests from their residents, the local city governments established a new organization named the “Conference on Radiation Countermeasures in the Tokatsu area (CRCT)” 3 months after the accident, and they officially surveyed the dose equivalent rates at public facilities such as schools and parks [9]. Information about the rates has been available on the websites of each local government office.

According to the results obtained from air- and car-borne surveys by the Japanese government measured in September 2011 [8], the Tokatsu area, excluding the north area of Noda City (#1 in Fig 1B) and the south area of Kamagaya City (#6 in Fig 1B), had dose equivalent rates (absorbed dose rate in air) roughly ranging from 0.2–0.5 μSv h^-1^ (267–668 nGy h^-1^, dose conversion factor: 0.748 Sv Gy^-1^ [10]). In the latest result measured in November 2015, Kashiwa and Abiko Cities had dose equivalent rates in the range of 0.1–0.2 μSv h^-1^ (134–267 nGy h^-1^), and the dose equivalent rates in the other four cities were below 0.1 μSv h^-1^ (134 nGy h^-1^) which is the minimum level [8]. A detailed dose rate distribution map to estimate the impact from the F1-NPP accident on Tokatsu area has not been obtained. Additionally, while the fixed-point observations for absorbed dose rate in air have been carried out by local governments, the general public is often ill-informed about the presence of natural radiation sources such as terrestrial gamma-rays and cosmic-rays and their contribution to the absorbed dose rates. According to the report from CRCT [11], as of December 2011, the average dose equivalent rate (absorbed dose rate in air) for the Tokatsu area measured with a CsI(Tl) portable scintillation survey meter was 0.18 ± 0.07 μSv h^-1^ (224 ± 99 nGy h^-1^) at 1 m above the ground surface; however, the measured values are mixed dose rates from the natural and artificial radionuclides.

Researchers at the National Institute of Radiological Sciences (NIRS) carried out a nationwide survey of absorbed dose rate in air from natural radiation in the 1960s–1970s [12]. In this survey, done well before the F1-NPP accident, measurements were made on school grounds in Noda (*n* = 1), Kashiwa (*n* = 2) and Matsudo (*n* = 3) Cities for the Tokatsu area, but measurements were not made in the other three cities. Sugino et al. [13] also measured the terrestrial gamma ray dose rate by fixed-point observation for the Kanto district which included Chiba Prefecture, but the whole Tokatsu area was not covered by the measurement points. Therefore, no accurate estimation has been made for the impact of the F1-NPP accident on the Tokatsu area. In this study, a car-borne survey for the whole Tokatsu area was carried out to make the detailed dose rate distribution map. Additionally, the gamma-ray pulse height distribution by the fixed-point observation was determined using a NaI(Tl) scintillation spectrometer to estimate the contribution ratio of artificial radionuclides for the dose rate.

## Materials and Methods

### Survey route

**Fig 2:**
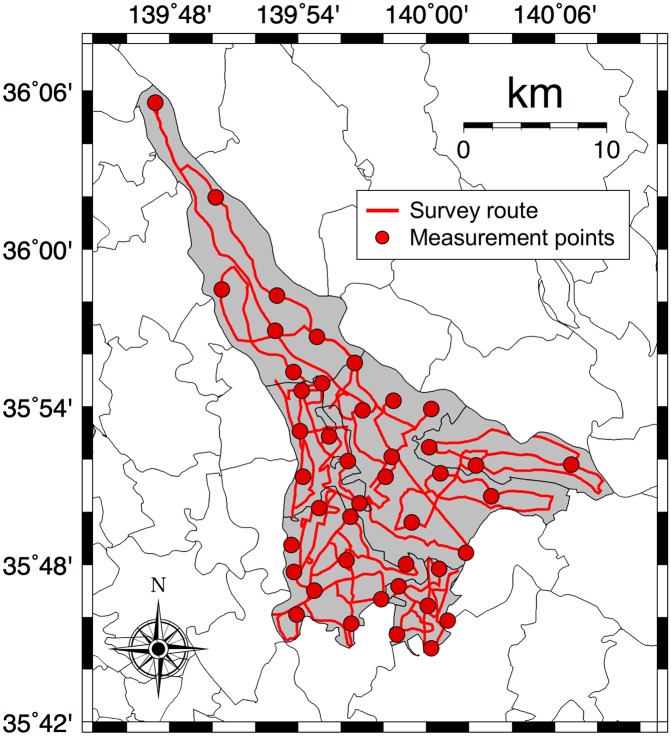
Survey routes for measuring the absorbed dose rates in air. A car-borne survey was carried out using a NaI(Tl) scintillation spectrometer in June 2015. Total distance traveled was 669 km. The fixed-point measurements outside the car were also carried out for 10 min at 43 locations.

The absorbed dose rates in air (nGy h^-1^) from both natural radionuclides (^40^K, ^238^U series and ^232^Th series) and artificial radionuclides (^134^Cs and ^137^Cs) were measured on June 5 and 10–14, 2015, in the Tokatsu area of Chiba Prefecture, Japan (Fig 1). The survey route is shown in Fig 2. Main roads including national routes (NRs) 6 and 16 were selected to the extent possible, primarily centered on residential areas. No expressways were included in this survey. The survey route was 669 km long. The weather condition was sunny or cloudy throughout the survey. This route map was drawn using the Generic Mapping Tools (GMT) created by Wessel and Smith [14].

### Car-borne survey

A car-borne survey technique is a convenient method for the evaluation of radiation dose in a wide area in a short period [15]. A 3-in × 3-in NaI(Tl) scintillation spectrometer (EMF211, EMF Japan Co., Osaka, Japan) with a global positioning system was used for the present car-borne survey. This spectrometer was positioned inside the car. Measurements of the counts inside the car were carried out every 30 s along the route. Latitude and longitude at each measurement point were measured at the same time as the gamma-ray count rates (50 keV– 3.2 MeV) were recorded. Car speed was kept around 40 km h^-1^. The photon peak of ^40^K (E_*γ*_ = 1.464 MeV) and ^208^Tl (E_*γ*_ = 2.615 MeV) was used for the gamma-ray energy calibration from the channel number and gamma-ray energy before the measurements. Measured count rates inside the car were corrected by multiplying them by a shielding factor to estimate the unshielded external dose rates. The shielding factor of the car body was estimated by making measurements inside and outside the car at 43 locations (Fig 2). Those measurements were recorded over consecutive 30-s intervals during a total recording period of 2 min. A shielding factor was obtained from the slope of the regression line in the relation between count rates inside and outside the car.

The gamma-ray pulse height distributions were also measured outside the car for 10 min at 43 locations (Fig 2). These observations were carried out on private land after obtaining specific permissions from the land owners and it was also confirmed that the field studies did not involve endangered or protected species. The NaI(Tl) scintillation spectrometer was positioned 1 m above the ground surface. Measured gamma-ray plus height distributions were then unfolded using a 22 × 22 response matrix for the estimation of absorbed dose rate in air. The detailed method has been reported by Minato [16]. These calculated dose rates were used to estimate the dose conversion factor (nGy h^-1^/cps) because it is difficult to obtain the photon peak for each gamma-ray energy in a 30-s measurement. In this study, the dose conversion factor was obtained from the slope of the regression line in the relation between corrected inside count rates and calculated absorbed dose rates in air, and inside count rates were multiplied by the dose conversion factor to convert them to external absorbed dose rate in air.

Based on all of the calculated external absorbed dose rates in air, the detailed dose distribution map in the Tokatsu area was plotted using GMT [14] and the plotted data on the map were interpolated using a minimum curvature algorithm. The calculated absorbed dose rates in air using a 22 × 22 response matrix method were separated as natural radionuclides (^40^K, ^238^U series and ^232^Th series) and artificial radionuclides (^134^Cs and ^137^Cs). In this study, the energy bins were set to 1.39–1.54 for ^40^K, 1.69–1.84 MeV and 2.10–2.31 MeV for ^214^Bi (^238^U series), 2.51–2.72 MeV for ^208^Tl (^232^Th series), 0.55–0.65 MeV and 0.75–0.85 MeV for ^134^Cs and 0.65–0.75 MeV for ^137^Cs to unfold the gamma-ray pulse height distribution, and the contribution ratio of artificial radionuclides for absorbed dose rate in air was observed in the spectra. These energy intervals for the bins were given by Minato [16]. Additionally, external effective dose (mSv) was estimated based on the measured absorbed dose rate in air.

## Results and Discussion

### Shielding and dose conversion factors

**Fig 3:**
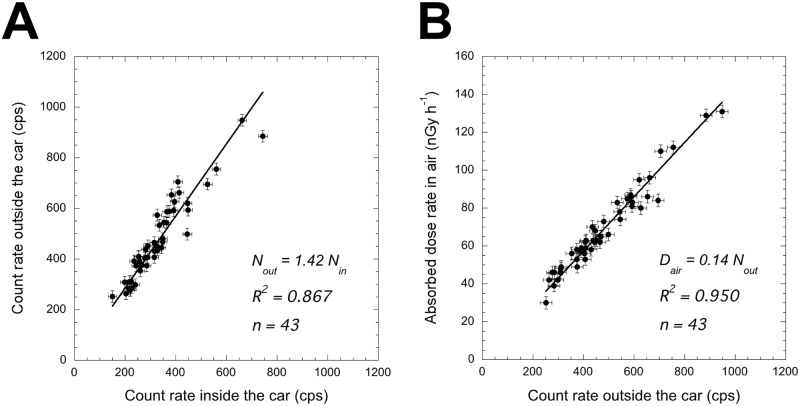
Correlation between count rates inside and outside the car (A) and between absorbed dose rate in air and count rates outside the car (B). The slopes of these regression lines were used as the shielding factor (1.42) and the dose conversion factor (0.14). The standard errors of regressions were shown as error bars.

The correlation between count rates inside and outside the car measured at 43 locations is shown in Fig 3A, and the shielding factor and standard uncertainty [17] were found to be 1.42 and 0.08, respectively. Although the shielding factor is influenced by the type of car, number of passengers and dosimeter position inside the car, this factor has been reported in previous reports as ranging from 1.3–1.9 [2, 5–7, 15, 18–20]. The presently obtained factor was in this range. Fig 3B shows the correlation between absorbed dose rate in air (nGy h^-1^) calculated using the 22 × 22 response matrix method and count rate outside the car (cps) (i.e., corrected count rate inside the car). The dose conversion factor and uncertainty were found to be 0.14 nGy h^-1^/cps and 0.01, respectively. The decision coefficients (*R*^*2*^) for shielding and dose conversion factors were 0.867 and 0.950, respectively (Fig 3A and 3B). A lower decision coefficient of the shielding factor was observed compared to previous measurements made in Aomori, Japan (*R*^*2*^ = 0.973, *n* = 73) [7], Kerala, India (*R*^*2*^ = 0.964, *n* = 34) [15] and Brunei (*R*^*2*^ = 0.97, *n* = 16) [21] which were places not contaminated by artificial radionuclides. It seemed that the lower decision coefficient might be affected by heterogeneously deposited artificial radionuclides.

Based on these results, the absorbed dose rates in air (*D*_*out*_) outside the car 1 m above the ground surface at each measurement point were calculated using Eq (1):

$$D_{out}=D_{in}\times 1.42\times 0.14$$ (1)

where *C*_*in*_ is count rate inside the car (cps) obtained by the measurements of the car-borne survey.

### Contribution ratio of artificial radionuclides

**Fig 4:**
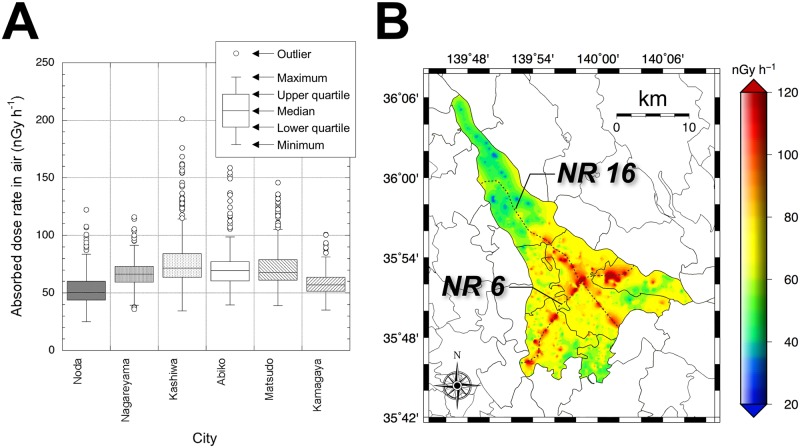
The distribution map of absorbed dose rate in air for the Tokatsu area. A minimum curvature algorithm was used for the data interpolation using the Generic Mapping Tools of Wessel and Smith [14]. This map was drawn using 2,165 data.

The statistically analyzed absorbed dose rates in air measured by the car-bone survey are shown in Fig 4A. The outliers were defined as: < lower quartile– 1.5 × distance from upper quartile to lower quartile (*IQD*) or > upper quartile + 1.5 × *IQD* (KaleidaGraph, Synergy Software, USA). The mean absorbed dose rate in air in the whole Tokatsu area was 68 ± 20 nGy h^-1^ (25–201 nGy h^-1^). The mean and range of absorbed dose rate in air of the six cities are shown in Table 1. The most contaminated city was Kashiwa, whereas the least contaminated was Noda. Absorbed dose rates in air observed from all radionuclides, natural radionuclides (^40^K, ^238^U series and ^232^Th series) and artificial radionuclides (^134^Cs and ^137^Cs) at 43 locations are shown in Table 2. The mean dose rates from natural and artificial radionuclides were 46 ± 10 nGy h^-1^ (24–82 nGy h^-1^) and 24 ± 19 nGy h^-1^ (0–78 nGy h^-1^), respectively. According to Abe et al. [12] the absorbed dose rates in air measured in the 1960s to 1970s in Noda, Kashiwa and Matsudo Cities were 55, 51 and 59 nGy h^-1^, respectively, and these values were higher than those of the present study. Because the pavement ratio has increased from 43% in 1973 to 91% in 2011, the shielding effect of terrestrial gamma-rays by asphalt has increased accordingly. Additionally, the effects from the nuclear tests performed in the 1950s to 1980s and Chernobyl NPP accident in 1986 cannot be ignored. According to reporting from the Meteorological Research Institute [22], the integrated deposited activity of ^137^Cs with decay for 1954 to before the F1-NPP accident was estimated to be 2 kBq m^-2^, whereas that for 2011 (i.e., after the F1-NPP accident) was 25 kBq m^-2^/y, resulting in a 12.5-fold difference. Although the above values are simple integrated deposited activities, it was estimated that the measured dose rate in this study included a few nano-gray contribution from the earlier events. The mean contribution ratio of artificial radionuclides to the absorbed dose rate in air measured 4 years after the F1-NPP accident in the six cities was 29% (9–50%). The authors previously obtained the contribution ratio of artificial radionuclides measured in December 2014 in metropolitan Tokyo’s 23 wards, located to the southwest from the Tokatsu area, as 13% (0–36%; *n* = 26) [19]. The degree of contamination in the Tokatsu area was 16% higher than that of Tokyo’s 23 wards.

**Table 1:** Absorbed dose rate in air in the Tokatsu area of Chiba Prefecture measured in 2015.

No.^a^	Municipality (City)	*n*	Absorbed dose rate in air (nGy h^-1^)			External effective dose (mSv y^-1^)
			Mean	SD	Range	
1	Noda	313	53	14	25–122	0.16
2	Nagareyama	327	67	12	36–116	0.20
3	Kashiwa	487	77	23	34–201	0.23
4	Abiko	181	73	22	40–159	0.22
5	Matsudo	570	73	18	39–146	0.22
6	Kamagaya	278	58	11	35–101	0.17

^a^ The numbers refer to the designations in Fig 1B.

**Table 2:** Absorbed dose rate in air from natural and artificial radionuclides in the Tokatsu area of Chiba Prefecture.

No.^a^	Municipality (City)	*n*	Absorbed dose rate in air (nGy h^-1^)									Contribution ratio of artificial radionuclides (%)
			All radionuclides			Natural radionuclides			Artificial radionuclides			
			Mean	SD	Range	Mean	SD	Range	Mean	SD	Range	
1	Noda	7	49	6	42–59	45	3	39–48	4	6	0–14	9.1
2	Nagareyama	7	84	22	49–112	54	16	32–82	29	15	17–62	35.3
3	Kashiwa	10	87	25	58–131	43	10	24–58	44	21	17–78	50.4
4	Abiko	3	61	5	56–65	48	3	45–50	13	7	6–21	21.1
5	Matsudo	10	73	16	53–96	47	7	32–58	26	11	8–39	35.6
6	Kamagaya	6	56	15	39–81	44	6	35–82	12	13	1–34	21.7

^a^ The numbers refer to the designations in Fig 1B.

### Distribution of absorbed dose rate in air in Tokatsu area

Fig 4B shows the distribution map of absorbed dose rate in air in the whole Tokatsu area measured in 2015. This map was drawn using 2,165 data. According to the air- and car-borne surveys during August 2011 to May 2012 [3], higher dose areas were observed for the southern area of Ibaraki Prefecture (Fig 1) and they subsequently extended toward the southwest direction (i.e., Tokatsu area). Thus, this shift might have influenced absorbed dose rate in the Tokatsu area. A heterogeneous distribution of absorbed dose rates in air was seen. Especially, higher absorbed dose rates in air of over 80 nGy h^-1^ were observed along NRs 6 and 16 that pass through Matsudo (#5 in Fig 1B), Kashiwa (#3 in Fig 1B) and Abiko (#4 in Fig 1B) Cities. The highest absorbed dose rate in air (i.e., 201 nGy h^-1^) was observed at Akebono-cho, Kashiwa City (Fig 1B) where these two routes cross each other. These higher absorbed dose rates in air seemed to be related to rates in parts of metropolitan Tokyo [19]. Especially, for Katsushika Ward (Fig 1B) where the highest absorbed dose rate in air in metropolitan Tokyo was observed, higher absorbed dose rates in air exceeding 70 nGy h^-1^ were measured in 2014 all along NR 6 and other main roads [5], and their tendency was similar to that of the present study. According to the results of the air-borne survey performed in September 2011 [8], a homogeneous dose rate distribution was observed for the Tokatsu area excluding Noda City. A similar result was also obtained from a car-borne survey performed by Andoh et al. [3]. The dose distributions measured in September 2011 [8] and in the present study showed different tendencies, homogeneous versus heterogeneous.

### Contamination of artificial radionuclides on asphalt surfaces

**Fig 5:**
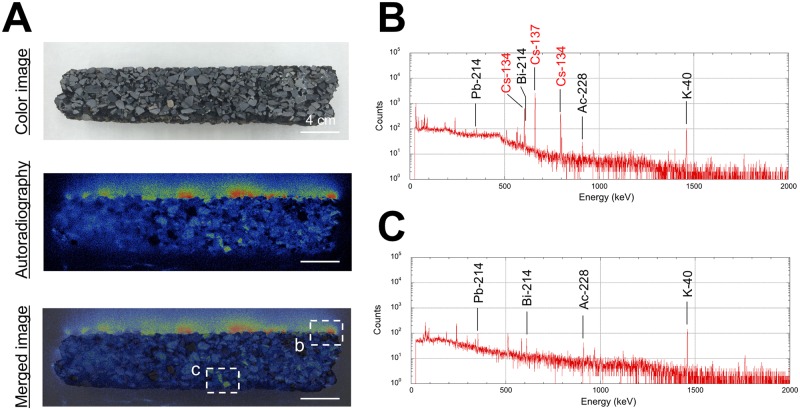
The color image, autoradiography image and merged image of a cross section of porous asphalt. The porous asphalt was collected in Akebono-cho, Kashiwa City (#3 in Fig 1B). The autoradiography image (A) was obtained by exposing a phosphor-imaging screen to beta and gamma rays emitted from an asphalt sample for 1 week. The energy spectra of the surface (B) and deep (C) layers of this asphalt sample as measured by a high-purity germanium semiconductor detector for 30,000 s.

For a more detailed evaluation, asphalt samples were collected from Akebono-cho (Fig 1B) which had the highest absorbed dose rate in air, and they were imaged using autoradiography. In this study, this sample (i.e., asphalt spoil) was received from Kumagai Gumi Co. Ltd. (Tokyo, Japan) with their permission in association with roadwork the company was carrying out. Fig 5A shows the color, autoradiography, and merged images of one asphalt sample. The autoradiography image was obtained by exposing a phosphor-imaging screen to beta- and gamma-rays emitted from an asphalt sample for 1 week and scanning the phosphor-imaging screen using a FLA-7000 scanner (Fujifilm Co., Ltd., Tokyo, Japan). Higher image intensities were observed in the upper 1 cm asphalt surface layer. The energy spectra of the surface and deep layers are shown in Fig 5B and 5C, respectively. Dominant peaks were detected for radiocesium, such as ^134^Cs (E_*γ*_ = 605 and 796 keV) and ^137^Cs (E_*γ*_ = 662 keV), and for natural radionuclides, such as ^40^K (E_*γ*_ = 1461 keV), ^228^Ac (E_*γ*_ = 911 keV), ^214^Pb (E_*γ*_ = 352 keV) and ^214^Bi (E_*γ*_ = 609 keV), from the asphalt surface layer, but no dominant peaks were detected for radiocesium (^134^Cs and ^137^Cs) from the deep layer. The authors collected samples of porous asphalt with a coarse aggregate diameter of more than 2.36 mm. This type asphalt has a high drainage function, resulting in its wide use recently for highways and main roads including NRs as show in Fig 4B to provide improved visibility for drivers in the rain. According to a supplier of such asphalt, it can be quickly clogged by dust depending on the amount of traffic. Thus, the deposited radiocesium remained within the 1 cm layer from the asphalt surface. Additionally, the deposited radiocesium was firmly attached to the dust particles near the asphalt surface [23]. The NRs 6 and 16 with road width of 20 m are heavily traveled roads (55,000 cars per day for each NR) compared to local roads, and it was expected that the amount of dust on the surface was extremely high. Thus, higher absorbed dose rates in air were observed along the NRs.

On the other hand, radiocesium deposited on the low porosity asphalt (diameters of fine aggregates of asphalt ranged from 0.075 mm ≤ D < 2.36 mm) which is utilized for local roads is easily washed out by rainfall compared to high porosity asphalt. The low porosity asphalt has a water repellency effect. Additionally, local roads tend to have a gentle center crown, and drainage ditches are placed along the sides of the roads to carry away the rainfall. Therefore, the low porosity asphalt surface has hardly any dust deposition compared to high porosity asphalt because of the difference in natural weathering processes on the road surface. Thus, the degree of radiocesium contamination on the low porosity asphalt surface was low. In fact, most of the radiocesium had been held in only the upper 1 mm layer of low porosity asphalt in the test performed in the 1990s on mechanical decontamination measures after the Chernobyl accident [23]. Thus, the heterogeneous dose distribution measured 4 years after the F1-NPP accident (Fig 4) was made depending on the difference of asphalt types and traffic volume (i.e., dust volume on the asphalt surface).

### External effective dose estimation

The external effective doses for the six cities of Tokatsu area were estimated using the following equation:

$$E=D_{out}\times DCF\times T\times \left(Q_{in}\times R+Q_{out}\right)\times 10^{-6}$$ (2)

where *E* is the external effective dose (mSv y^-1^), *D*_*out*_ is the average absorbed dose rate in air (nGy h^-1^), *DCF* is dose conversion factor from the dose rate to the external effective dose for adults (0.748 ± 0.007 Sv Gy^-1^) [10], *T* is 8,760 h (24 h × 365 d), and *Q*_*in*_ and *Q*_*out*_ are indoor (0.9) and outdoor (0.1) occupancy factors [24], respectively. *R* is the ratio of indoor dose rate to outdoor dose rate (0.4) for 1- and 2-story wooden houses [25]. The estimated external effective doses (mSv y^-1^) for the six cities are shown in Table 1. The average value for Tokatsu area was 0.20 mSv y^-1^. This value was 60% of the Japan average before the F1-NPP accident (0.33 mSv y^-1^) [26] and 42% of the worldwide average (0.48 mSv y^-1^) [27]. In addition, the average value for Tokatsu area was only 9% of the reported annual medical exposure dose from CT examinations for Japan which is 2.3 mSv y^-1^/person [28].

### Environmental half-life on asphalt pavements

**Fig 6:**
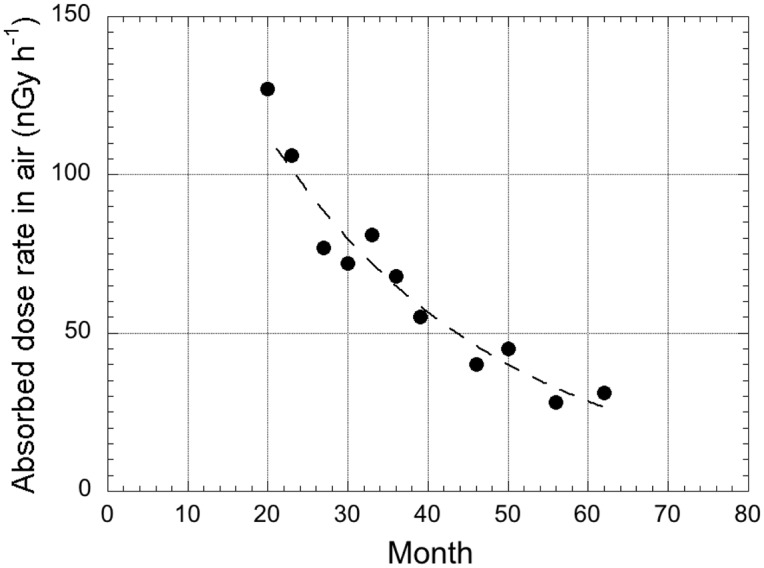
Transition of representative absorbed dose rate in air between October 2012 and April 2014 at Akebono-cho, Kashiwa City (Fig 1B). This figure was drawn using data published by the Kashiwa City Office [29]. These data were measured by the car-borne survey technique using a CsI(Tl) scintillation spectrometer.

The dose equivalent rate for all of Kashiwa City has been regularly observed since October 1, 2012 by the Kashiwa City Office using a CsI(Tl) scintillation spectrometer (Mobile G-DAQ, Keisokugiken Co., Tochigi, Japan) [29]. Fig 6 shows the change of absorbed dose rate in air at Akebono-cho (Fig 1B). The dose conversion factor used was 0.784 Sv Gy^-1^ [10] for converting to the absorbed dose rate in air. The absorbed dose rate in air from artificial radionuclides was calculated by subtracting background absorbed dose rate in air observed in this study (i.e., 43 nGy h^-1^ in Table 2). The origin of the horizontal axis was set to March 21, 2011 when the F1-NPP radioactive plume was observed around the Tokatsu area [30]. Here, the decay constant and environmental half-life by artificial radionuclides were calculated with the following equation to estimate the change of absorbed dose rate in air in the future:

$$D=D_{L}\left[\text{exp}\left(-\lambda_{L}t\right)\right],\;T_{environ}=0.693/\lambda_{L}$$ (3)

where *D* is the absorbed dose rate in air by artificial radionuclides, *D*_*L*_ is the initial absorbed dose rate in air due to long half-life radionuclides (^134^Cs and ^137^Cs), *λ*_*L*_ is the decay constant, *t* is the elapsed years after the date that the radioactive plume reached the Tokatsu area and *T*_*environ*_ is environmental half-life (year). In the present study, the environmental half-life was defined as “apparent half-life” to distinguish it from physical half-life. Thus, the calculated half-life in the above included the effect of physical half-life and mechanical wear of the asphalt surface. In some previous reports, this apparent half-life was described as the ecological half-life or environmental half-life [31, 32]. As a result, *D*_*L*_, *λ*_*L*_ and *T*_*environ*_ were 223 nGy h^-1^, 0.034 and 1.7 y, respectively. That environmental half-life was shorter than the physical half-life of ^137^Cs. In Katsushika Ward (Fig 1B), near the Tokatsu area, the environmental half-life 3 months after the accident was estimated to be 1.9 y based on changes of the absorbed dose rates in air at 1 m above the ground surface [20]. In addition, in the Chernobyl accident, the environmental half-life of ^137^Cs was calculated to be 3–4 years for lichen species [31, 33]. The calculated environmental half-life at Kashiwa City was shorter than the values of this report because surface contamination on asphalt is easily washed away by rainfall compared to bare ground or lichen-covered areas. Additionally, the environmental half-life on asphalt might be highly dependent on the traffic volume (which relates to the amount of dust) and the type of asphalt.

### Combined relative standard uncertainty for the car-borne survey in Tokatsu area

The standard uncertainties of the one-time measurement (30 s) can be calculated from the measured value. The obtained range of counts inside the car was 3600–28770 (counts per 30 s) in the present study. The standard uncertainty depending on measured counts was calculated to be 120–339 counts. The range of relative standard uncertainty for the 30-s measurements was also calculated to be 1.2–3.3%. Here, the relative standard uncertainties for the shielding factor, dose conversion factor, traceability of the dose rate (calibrated by Pony Industry Co., Ltd., Osaka, Japan), and the dose calculation procedure by the response matrix method (software developed by EMF Japan Co., Osaka, Japan) were given as 7.5%, 0.5%, 4.1% (*k* = 2), and 5.0%, respectively. The maximum combined relative standard uncertainty of the estimate absorbed dose rate in air in this study was calculated to be 11.6%.

## Conclusion

The car-borne survey with a NaI(Tl) scintillation spectrometer was carried out for the Tokatsu area, located in northwestern Chiba Prefecture, Japan, to make the detailed distribution map of absorbed dose rate in air 4 year after the F1-NPP accident. While the absorbed dose rate in air just after the accident had shown a homogeneous distribution in the Tokatsu area, it was now a heterogeneous distribution. Higher absorbed dose rates in air of over 80 nGy h^-1^ were observed along NRs 6 and 16. The type of asphalt and traffic volume strongly affected the dose distribution. Radiocesium (^134^Cs and ^137^Cs) was deposited within a 1 cm layer from the asphalt surface. The environmental half-life of radiocesium on asphalt was estimated to be 1.7 y. The means absorbed dose rate in air from radiocesium and the contribution ratio of radiocesium to the absorbed dose rate in air for Tokatsu area were 24 ± 19 nGy h^-1^ and 29% (9–50%), respectively. It was estimated that this measured dose rate included a few nano-gray contribution from the nuclear tests performed in the 1950s to 1980s and the Chernobyl NPP accident in 1986. The external effective dose calculated based on dose rate from natural and artificial radionuclides for Tokatsu area was 0.20 mSv y^-1^ which is 42% of the world wide average (0.48 mSv y^-1^).
